# What are risk factors for subsequent fracture after vertebral augmentation in patients with thoracolumbar osteoporotic vertebral fractures

**DOI:** 10.1186/s12891-021-04946-7

**Published:** 2021-12-13

**Authors:** Zhi Chen, Chenyang Song, Min Chen, Hongxiang Li, Yusong Ye, Wenge Liu

**Affiliations:** 1 Department of Orthopedics Surgery, Fujian Medical University Union Hospital, Fuzhou, 350001 Fujian China; 2 Department of Radiology, Fujian Medical University Union Hospital, Fuzhou, 350001 Fujian China; 3 Department of Orthopedics Surgery, Fuqing Affiliated Hospital of Fujian Medical University, Fuzhou, 350001 Fujian China

**Keywords:** Osteoporotic vertebral compression fracture, Vertebroplasty, Kyphoplasty, Refracture, Paraspinal muscle, Spine sagittal alignment, Risk factor

## Abstract

**Background:**

Due to its unique mechanical characteristics, the incidence of subsequent fracture after vertebral augmentation is higher in thoracolumbar segment, but the causes have not been fully elucidated. This study aimed to comprehensively explore the potential risk factors for subsequent fracture in this region.

**Methods:**

Patients with osteoporotic vertebral fracture in thoracolumbar segment who received vertebral augmentation from January 2019 to December 2020 were retrospectively reviewed. Patients were divided into refracture group and non-refracture group according to the occurrence of refracture. The clinical information, imaging findings (cement distribution, spine sagittal parameters, degree of paraspinal muscle degeneration) and surgery related indicators of the included patients were collected and compared.

**Results:**

A total of 109 patients were included, 13 patients in refracture group and 96 patients in non-refracture group. Univariate analysis revealed a significantly higher incidence of previous fracture, intravertebral cleft (IVC) and cement leakage, greater fatty infiltration of psoas (FI_PS_), fatty infiltration of erector spinae plus multifidus (FI_ES + MF_), correction of body angle (BA), BA restoration rate and vertebral height restoration rate in refracture group. Further binary logistic regression analysis demonstrated previous fracture, IVC, FI_PS_ and BA restoration rate were independent risk factors for subsequent fracture. According to ROC curve analysis, the prediction accuracy of BA restoration rate was the highest (area under the curve was 0.794), and the threshold value was 0.350.

**Conclusions:**

Subsequent fracture might cause by the interplay of multiple risk factors. The previous fracture, IVC, FI_PS_ and BA restoration rate were identified as independent risk factors. When the BA restoration rate exceeded 0.350, refractures were more likely to occur.

**Supplementary Information:**

The online version contains supplementary material available at 10.1186/s12891-021-04946-7.

## Background

With the aging of population, osteoporotic vertebral compression fracture (OVCF) has become a major public health issue, affecting millions of patients worldwide [1]. Vertebral augmentation, with the characteristics of rapid pain relief and function rehabilitation, has been widely accepted for the treatment of symptomatic OVCF [2]. However, more and more studies suggested this procedure might accelerate or facilitate subsequent fractures, which lead to renewed pain, reduced daily activity and repeated treatment [3, 4].

Due to its unique anatomical location and mechanical characteristics, thoracolumbar segment has a higher incidence of subsequent fracture after vertebral augmentation, but the causes have not been fully elucidated. While scholars confirmed some risk factors, other factors, such as intravertebral cleft (IVC), cement distribution and leakage, correction of kyphotic deformity, were controversial to date. Furthermore, more recent studies suggested paraspinal muscle atrophy might play a role in chronic low back pain and lumbar degenerative diseases. Whereas the exact association between paraspinal muscle degeneration with subsequent fracture after vertebral augmentation remained largely unknown. In this context, we conducted this study to comprehensively evaluate the potential risk factors for subsequent fracture in thoracolumbar segment, including the effect of paraspinal muscles.

## Methods

### Study participants

This retrospective study was conducted in the orthopedic department of two hospitals, vertebral augmentation procedures were performed by four senior surgeons via bilateral transpedicular approach according to standard procedures. Patients with symptomatic OVCF in thoracolumbar segment (T10-L2) who treated with vertebral augmentation from January 2019 to December 2020 were retrospectively enrolled. The inclusion criteria were as follows: (1) patients>65 years old with single or multiple level acute OVCF in the thoracolumbar segment. (2) patients received percutaneous vertebroplasty (PVP) or percutaneous kyphoplasty (PKP) treatment. (3) patients with at least 6 months follow-up data. And the exclusion criteria included: (1) patients with OVCF in other segments. (2) patients received other treatments. (3) fractures caused by severe trauma or pathological fractures due to tumor, infection or bone metabolic disease. (4) patients with previous spinal surgery. (5) patients with incomplete follow-up data.

### Data collection and image analysis

**Fig. 1:**
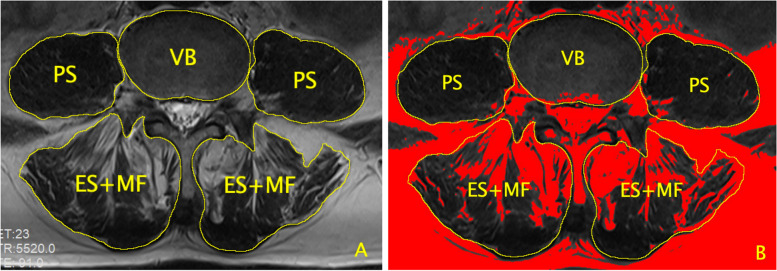
Measurement methods of CSA and FI of paraspinal muscle (psoas (PS), erector spinae plus multifidus (ES + MF)) and CSA of vertebral body (VB)

The included patients were assigned into refracture group and non-refracture group according to the occurrence of refracture during the follow-up. For each included patient, the following clinical information were collected: age, gender, previous fracture history, number and level of primary and refracture vertebrae, surgical technique, duration of follow-up. In addition, the presence of IVC, cement distribution (12 scores method) [5] and leakage, preoperative anterior height of fractured vertebrae and intact adjacent vertebrae above and below it, postoperative anterior height of cemented vertebrae, preoperative body angle (pre-BA), Cobb’s angle (pre-CA), thoracolumbar kyphosis (pre-TLK), lumbar lordosis (pre-LL) and postoperative body angle (post-BA), the cross-sectional area (CSA) of vertebral body, the CSA and fatty infiltration (FI) of bilateral paraspinal muscles (psoas (PS) and erector spinae plus multifidus (ES + MF)) (Fig. 1) at the superior endplate of L4 on preoperative T2-weighted axial image were obtained [6]. (The CSA and FI of paraspinal muscles, the vertebral height were measured using Image J V1.8, National Institutes of Health, USA, and the angles were measured using DICOM viewer Weasis, V1.2.4, Weasis Team) Of note, the vertebral height, pre-BA, pre-CA, post-BA and cement distribution were not collected in patients with multiple fractures. Based on the results of above parameters, the relative CSA (r-CSA) of paraspinal muscles (r-CSA_PS_ and r-CSA_ES + MF_), vertebral compression rate, vertebral height restoration rate, BA restoration rate and correction of BA were also calculated. (The methods used to measure and calculate these parameters were demonstrated in Supplemental Table 1 and Fig. 1, Supplemental Fig. 1).

After reaching an agreement, the above parameters were independently measured and calculated by a spine surgeon and a radiologist. The interobserver reliability was assessed via the intraclass correlation coefficient (ICC), and the result showed excellent.

### Statistical analysis

All statistical analyses were conducted using statistics software SPSS 23.0, and significant differences were indicated when *p* < 0.05. The Chi-square test (for categorical data), the Student’s t-test (for normally distributed data) and the Mann-Whitney U-test (for non-normally distributed data) were used to compare the difference between the two groups. Variables with a statistical difference were entered into binary logistic regression analysis to identify independent risk factors, and ROC curve was used to predict the critical value.

## Results

### Demographic characteristics and imaging findings

Based on the inclusion and exclusion criteria, a total of 109 patients were included in this study, the duration of follow-up was 17.53 ± 6.47 month. There were 13 patients in refracture group (age: 78.85 ± 7.18) and 96 patients in non-refracture group (age: 76.51 ± 7.27). Univariate analysis revealed no significant differences in age, sex, number of fracture (single or multiple), surgical technique (PVP or PKP), cement distribution, vertebral compression rate, r-CSA_PS_, r-CSA_ES + MF_, pre-BA, pre-CA, pre-TLK, pre-LL and post-BA. But the results showed significantly higher incidence of previous fracture (*P* = 0.032), IVC (*P* = 0.022) and cement leakage (*P* = 0.011), greater FI_PS_ (*P* = 0.015), FI_ES + MF_ (*P* = 0.029), correction of BA (*P* = 0.004), vertebral height restoration rate (*P* = 0.018) and BA restoration rate (*P* = 0.002) in refracture group. (Table 1).

**Table 1:** Demographic characteristics and imaging findings of included patients

Parameters	Refracture(*n* = 13)	Non-refracture(*n* = 96)	*P*-value
Age, Mean ± SD	78.85 ± 7.18	76.51 ± 7.27	0.279
Sex (Male, n(%))	4 (30.8%)	27 (28.1%)	0.843
Previous fracture history, n(%)	4 (30.8%)	7 (7.3%)	**0.032**
Single/Multiple fracture (Single, n(%))	11 (84.6%)	88 (91.7%)	0.752
PVP/PKP (PVP, n(%))	7 (53.9%)	45 (46.9%)	0.637
Intravertebral cleft, n(%)	3 (23.1%)	3 (3.1%)	**0.022**
Cement leakage, n(%)	12 (92.3%)	53 (55.2%)	**0.011**
Vertebral compression rate, Mean ± SD	0.73 ± 0.14	0.72 ± 0.15	0.796
r-CSA_ES + MF_, Mean ± SD	1.08 ± 0.27	1.10 ± 0.28	0.806
r-CSA_PS_, Mean ± SD	0.45 ± 0.16	0.50 ± 0.48	0.684
FI_ES + MF_, Mean ± SD	0.40 ± 0.11	0.32 ± 0.13	**0.029**
FI_PS_, Mean ± SD	0.14 ± 0.08	0.08 ± 0.05	**0.015**
Pre-BA, Mean ± SD	13.87 ± 5.90	13.96 ± 5.89	0.962
Pre-CA, Mean ± SD	14.87 ± 9.98	16.56 ± 8.95	0.562
Pre-TLK, Mean ± SD	20.95 ± 13.28	23.47 ± 11.96	0.483
Pre-LL, Mean ± SD	39.96 ± 11.27	40.03 ± 13.53	0.985
Post-BA, Mean ± SD	7.90 ± 6.05	10.48 ± 4.98	0.118
Correction of BA, Mean ± SD	5.97 ± 2.91	3.49 ± 2.56	**0.004**
BA restoration rate, Mean ± SD	0.49 ± 0.23	0.24 ± 0.24	**0.002**
Vertebral height restoration rate, Mean ± SD	0.13 ± 0.10	0.08 ± 0.07	**0.018**
Cement distribution, Mean ± SD	10.27 + 1.40	10.20 + 1.63	0.886

### Binary logistic regression and ROC curve analysis

Based on the results of univariate analysis between the two groups, all statistically significant variables (previous fracture, IVC, cement leakage, FI_PS_, FI_ES + MF_, correction of BA, vertebral height restoration rate, BA restoration rate) were included in the binary logistic regression analysis. Among these variables, we found that previous fracture, IVC, FI_PS_ and BA restoration rate (all *P* < 0.05) were independent risk factors for subsequent fracture. (Table 2).

**Table 2:** Outcome of binary logistic regression analysis

Risk factor	B	*P*-value	OR	95% confidence of interval of OR	
				Lower bound	Upper bound
Previous fracture history	2.164	**0.031**	8.71	1.22	62.05
Intravertebral cleft	3.070	**0.009**	21.53	2.15	215.95
FI_PS_	14.946	**0.015**	3,096,757.65	17.93	5.35E+ 11
BA restoration rate	4.718	**0.005**	111.92	4.11	3045.57

*OR* Odds ratio, *FI*_*PS*_ Fatty infiltration of psoas, *BA* Body angle

**Fig. 2:**
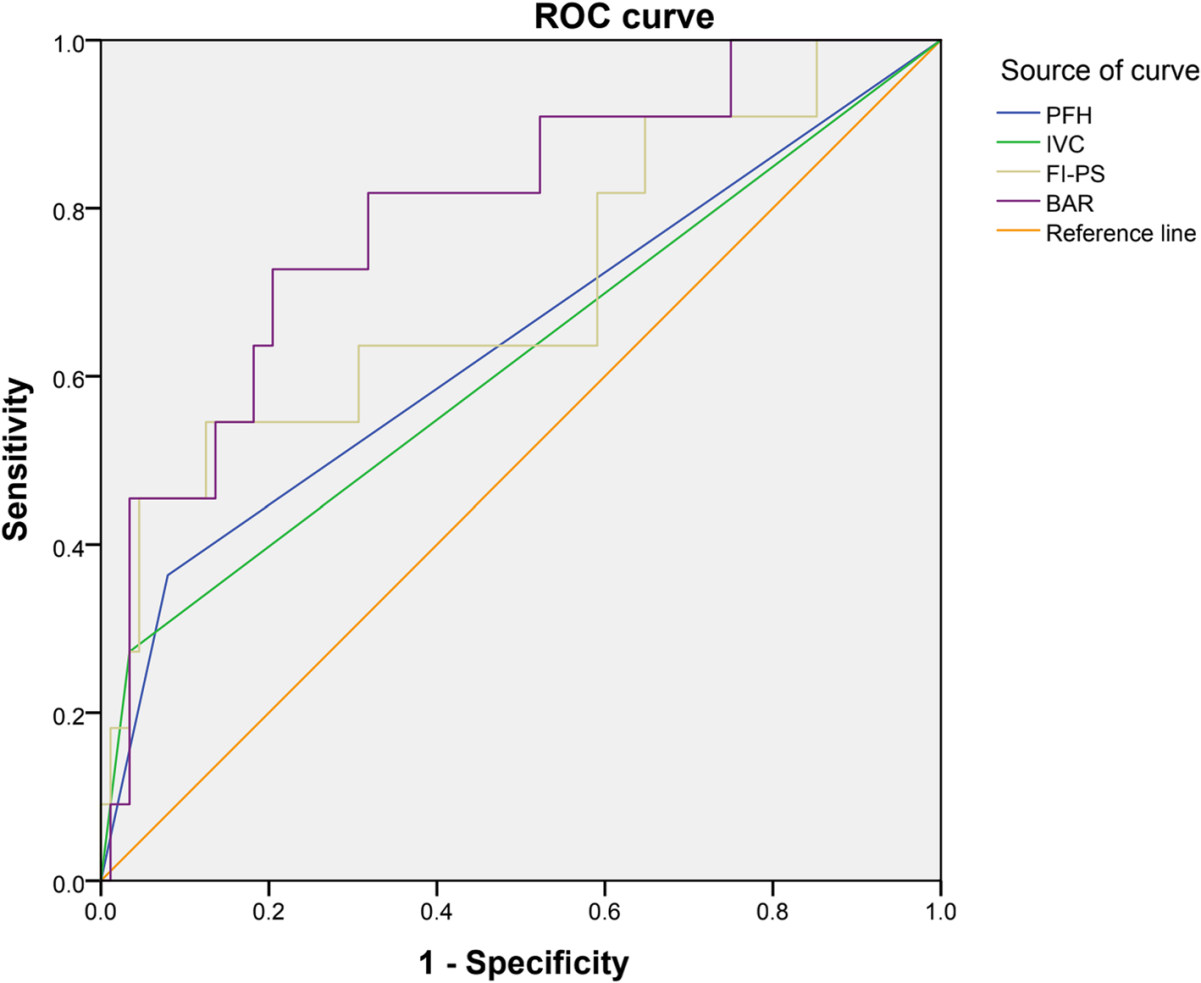
ROC curve

The ROC curves were used to further determine the degree of influence of each risk factor, and the results showed the prediction accuracy of BA restoration rate was the highest (area under the curve was 0.794). By calculating the threshold values, we found that patients would be more likely to suffer from subsequent fracture after surgery when the BA restoration rate was>0.350. (Fig. 2, Table 3).

**Table 3:** Area under the curve

Parameter	Area	Standard deviation	*P*-value	Asymptotic 95% Confidence Interval	
				Lower Bound	Upper Bound
PFH	0.642	0.100	0.126	0.446	0.838
IVC	0.619	0.102	0.198	0.420	0.818
FI_PS_	0.705	0.094	0.027	0.520	0.889
BAR	0.794	0.073	0.002	0.651	0.937

*PFH* Previous fracture history, *IVC* Intravertebral cleft, *FI*_*PS*_ Fat infiltration of psoas, *BAR* Body angle Restoration rate

## Discussion

Subsequent fracture is a serious complication following vertebral augmentation, which carries great burden on the patients and society. In view of the high incidence of subsequent fracture in thoracolumbar segment, it is imperative to identify the risk factors and develop targeted prevention strategies. After comprehensively evaluating the spine sagittal parameters, characteristics of paraspinal muscles and surgery related indicators, we found multiple factors were correlated with subsequent fracture after vertebral augmentation.

Several studies demonstrated previous fracture was an indirect reflection of patient’s bone quality [7, 8]. In a retrospective study, Ji et al. found that previous fracture history was significantly correlated with subsequent fracture following primary OVCF [8]. In another study, Lindsay et al. reported the risk of subsequent fracture was twofold higher after a non-spinal fracture and four times greater following a spinal fracture [7]. Our finding was consistent with previous work, patients with previous fracture were also the target population for the prevention of subsequent fracture after vertebral augmentation.

The presence of IVC is common in OVCF patients, especially in the thoracolumbar segment [9]. Although the relationship between IVC and subsequent fracture has received much attention, the results remain controversial [10, 11]. In a retrospective cohort study, Li et al. reported no significant difference in the incidence of subsequent fracture between patients with or without IVC [12]. Conversely, Kim and Yu found a significantly higher incidence of IVC in patients with subsequent fracture [11, 13]. Similar result was also observed in our study. It was likely that IVC indicated poorer blood supply and higher risk of cement leakage [14, 15], which might account for the increased risk of subsequent fracture.

Cement leakage was relatively common but always asymptomatic [16]. Some scholars held the view that cement leakage was generally no clinical significance as they did not find an association linking cement leakage and subsequent fracture [17, 18]. On the contrary, Bae et al. revealed a significantly higher incidence of cement leakage in refracture group [19]. Rho et al. suggested cement leakage was a primary predicting factor of subsequent fracture [20]. Moreover, Komemushi et al. indicated the risk of subsequent fracture was 4.6 times higher in patients with cement leakage than those without [21]. Our study again highlighted efforts should be made to reduce the incidence of cement leakage during surgery.

The importance of paraspinal muscles was once disregarded. Until recently, the critical role of paraspinal muscles in maintaining spinal stability and alignment was gradually elucidated. Some studies indicated there might be an association linking paraspinal muscle atrophy with etiology and healing of OVCF [6, 22, 23]. The finding reported by Deng et al. that postoperative low back muscle exercise could significantly reduce refracture risk, which suggested the protective role of paraspinal muscles [24]. In a multicenter cohort study of 153 patients with OVCF who received conservative treatment, Habibi et al. found that greater FI but not CSA of the paraspinal muscle was significantly associated with the occurrence of subsequent fracture [6]. Our finding further demonstrated greater FI was also an important risk factor for subsequent fracture following vertebral augmentation. The underlying mechanism has not yet been fully elucidated, some researchers hypothesized that fat infiltration of paraspinal muscles might reduce the muscle contractility and strength, which led to sagittal imbalance and increased stress on vertebral structures [25].

Restoring vertebral height and body angle to their original states were once considered to be the most desirable outcome [26]. However, studies on the subject reported controversial results. While Ning and colleagues did not detect a connection between vertebral height restoration rate and subsequent fracture [18]. Kim and Yoo et al. noted greater vertebral height restoration rate contributed significantly to the risk of subsequent fracture after percutaneous vertebroplasty [27, 28]. In terms of body angle correction, Takahashi et al. observed the correction degree was significantly greater in the refracture group than in the non-refracture group [29]. Similarly, Lin et al. reported that greater correction significantly increased the risk of refracture [30]. Our study confirmed again, too much correction of the vertebral height and body angle might lead to higher risk of subsequent fracture. Some researchers proposed that overcorrection of vertebral height and body angle would increase paravertebral soft tissue tension, which in turn increased mechanical load on the already weakened vertebrae [26].

In light of previous researches and our own, it is possible that the occurrence of subsequent fracture is generally not caused by a single risk factor, but rather by the interplay of multiple risk factors. Therefore, in patients with above mentioned risk factors, cement leakage and excessive correction should be avoided, and preventive measures should be taken, such as patient education, low back muscle exercise and anti-osteoporotic treatment.

This study had some limitations. Firstly, it was a two-center study, the surgeries were performed by different surgeons, and anti-osteoporotic regimes were not completely consistent, which might influence the outcomes. Secondly, other potential parameters, such as postoperative physical activity, smoking and drinking status, were not evaluated. Thirdly, it was a retrospective study and the number of patients was limited. Therefore, further studies are needed to verify our findings.

## Conclusion

Our study suggested the occurrence of subsequent fracture was caused by the interplay of multiple risk factors. The previous fracture, IVC, FI_PS_ and BA restoration rate were identified as independent risk factors. When the BA restoration rate exceeded 0.350, subsequent fracture was more likely to occur. Surgeons should adequately evaluate the above risk factors before surgery, and develop targeted prevention and treatment strategies to reduce this complication.

## Supplementary Information

**Additional file 1: Supplemental Table 1.** The measuring and calculating methods of cement distribution, spinal sagittal alignment, paraspinal muscle, vertebral height and body angle restoration rate.

**Additional file 2: Supplemental Figure 1.** Measurement methods of cement distribution (A and B), vertebral height (C) and spinal sagittal alignment (D and E).

## Data Availability

Data available on reasonable request.

## Abbreviations

IVC: Intravertebral cleft
BA: Body angle
FI: Fatty infiltration
OVCF: Osteoporotic vertebral compression fracture
CA: Cobb’s angle
TLK: Thoracolumbar kyphosis
LL: Lumbar lordosis
CSA: Cross-sectional area
PS: Psoas
ES: Erector spinae
MF: Multifidus
ICC: Intraclass correlation coefficient
