# Supplementary material for: What are risk factors for subsequent fracture after vertebral augmentation in patients with thoracolumbar osteoporotic vertebral fractures
Source: BMC Musculoskelet Disord. 2021 Dec 13;22:1040. doi: 10.1186/s12891-021-04946-7 (PMC8670201; doi:10.1186/s12891-021-04946-7)
Supplement: Supplementary file 1 — Additional file 1: Supplemental Table 1. The measuring and calculating methods of cement distribution, spinal sagittal alignment, paraspinal muscle, vertebral height and body angle restoration rate. [file 12891_2021_4946_MOESM1_ESM.docx]

| **Supplemental Table 1. The measuring and calculating methods** | |  |
| --- | --- | --- |
| **Parameters** | | **Methods** |
| **IVC** | | The finding of signal loss (gas-containing space) or hyperintensity (fluid aggregation) on preoperative sagittal T2- weighted images. |
| **Pre-BA** | | The angle between the upper and lower endplate of the fractured vertebrae. (Supplemental Figure 1. D) |
| **Pre-CA** | | The Cobb angle between the superior endplate of the upper vertebrae and the inferior endplate of the lower vertebrae. (Supplemental Figure 1. D) |
| **Pre-TLK** | | The Cobb angle between the T10 upper endplate and L2 lower endplate. (Supplemental Figure 1. E) |
| **Pre-LL** | | The Cobb angle between the L1 upper endplate and S1 lower endplate. (Supplemental Figure 1. E) |
| **CSA_muscle_** | | Area within the fascial border of the muscle. (Figure 1. A) |
| **CSA_vertebrae_** | | Area within the border of the vertebral body. (Figure 1. A) |
| **r-CSA** | | CSA_muscle_/CSA_vertebrae_. |
| **FI** | | The ratio of the fat signal divided by the CSA of the muscle. (Figure 1. B) |
| **Vertebral compression rate** | | $\frac{\text{preoperative anterior height of fractured vertebrae}}{\text{average anterior height of adjacent }\text{intact }\text{vertebra}\text{e}}$ |
| **Vertebral height restoration rate** | | $\frac{\text{postoperative anterior height of cemented vertebrae -preoperative anterior height of fractured vertebrae}}{\text{average anterior height of adjacent intact vertebrae}}$ |
| **Post-BA** | | The angle between the upper and lower endplate of the cemented vertebrae. |
| **Correction of BA** | | Postoperative BA- Preoperative BA. |
| **BA restoration rate** | | $\frac{\text{postoperative BA-preoperative BA}}{\text{preoperative BA}}$ |
| **Cement leakage** | | Any cement appears in the space outside the vertebral body. |
| **Cement distribution** (N=4+4+2+2) | | Vertebrae are divided into four quadrants based on the anterior-posterior and lateral positions. Quadrants are counted when the cement filling exceeds half of the quadrant. when the cement contacts the superior or inferior endplate of the vertebrae in the lateral plane, or if the cement crosses the midline on the anterior-posterior or lateral plane, then it is considered as an independently effective quadrant. (Supplemental Figure 1. A and B) |
|  | |  |
