# Supplementary material for: What are risk factors for subsequent fracture after vertebral augmentation in patients with thoracolumbar osteoporotic vertebral fractures
Source: BMC Musculoskelet Disord. 2021 Dec 13;22:1040. doi: 10.1186/s12891-021-04946-7 (PMC8670201; doi:10.1186/s12891-021-04946-7)
Supplement: Supplementary file 2 — Additional file 2: Supplemental Figure 1. Measurement methods of cement distribution (A and B), vertebral height (C) and spinal sagittal alignment (D and E). [file 12891_2021_4946_MOESM2_ESM.zip › Supplemental Figure legend.docx]

**Supplemental Figure 1.** Measurement methods of cement distribution (A and B), vertebral height (C) and spinal sagittal alignment (D and E).
